# Transcriptome Profiles Using Next-Generation Sequencing Reveal Liver Changes in the Early Stage of Diabetes in Tree Shrew (*Tupaia belangeri chinensis*)

**DOI:** 10.1155/2016/6238526

**Published:** 2016-03-16

**Authors:** Xiaoyun Wu, Haibo Xu, Zhiguo Zhang, Qing Chang, Shasha Liao, Linqiang Zhang, Yunhai Li, Dongdong Wu, Bin Liang

**Affiliations:** ^1^Key Laboratory of Animal Models and Human Disease Mechanisms of the Chinese Academy of Sciences and Yunnan Province, Kunming Institute of Zoology, Chinese Academy of Sciences, Kunming, Yunnan 650223, China; ^2^Key Laboratory of Puer Tea Science, Ministry of Education, Yunnan Agricultural University, Kunming, Yunnan 650201, China; ^3^State Key Laboratory of Genetic Resources and Evolution, Kunming Institute of Zoology, Chinese Academy of Sciences, Kunming, Yunnan 650223, China; ^4^School of Life Sciences, Anhui University, Hefei, Anhui 230601, China; ^5^Kunming College of Life Science, University of Chinese Academy of Sciences, Kunming, Yunnan 650204, China

## Abstract

Determining the liver changes during the early stages of diabetes is critical to understand the nature of the disease and development of novel treatments for it. Advances in the use of animal models and next-generation sequencing technologies offer a powerful tool in connection between liver changes and the diabetes. Here, we created a tree shrew diabetes model akin to type 1 diabetes by using streptozotocin to induce hyperglycemia and hyperlipidemia. Using RNA-seq, we compiled liver transcriptome profiles to determine the differentially expressed genes and to explore the role of hyperglycemia in liver changes. Our results, respectively, identified 14,060 and 14,335 genes in healthy tree shrews and those with diabetes, with 70 genes differentially expressed between the two groups. Gene orthology and KEGG annotation revealed that several of the main biological processes of these genes were related to translational processes, steroid metabolic processes, oxidative stress, inflammation, and hypertension, all of which are highly associated with diabetes and its complications. These results collectively suggest that STZ induces hyperglycemia in tree shrew and that hyperglycemia induced oxidative stress led to high expression of aldose reductase, inflammation, and even cell death in liver tissues during the early stage of diabetes.

## 1. Introduction

Decades of research into diabetes mellitus, a disease characterized by chronic hyperglycemia in the blood resulting from insulin resistance or insulin deficiency, paint a grim picture for the next 20 years. In 2010, 258 million adults had diabetes, but, despite ongoing searches for treatments paired with prevention efforts, that number is still expected to rise to nearly 439 million people by 2030, about 7.7% of the global adult population [1]. The seriousness of increasing diabetes is underscored by chronic hyperglycemia being among the most important factors involved in the complications and organ injuries that accompany diabetes, including cardiovascular disease, kidney disease, neuropathy, blindness, and liver injury, all of which are major risk factors for morbidity and mortality [2].

Given the connections between diabetes, chronic hyperglycemia, and their comorbidities, researchers have increasingly focused on exploring the liver, the primary organ involved in glucose metabolism and regulation, and the major target organ of insulin action. Previous reports found a heightened prevalence of liver diseases among diabetic patients to the extent that liver disease ranks as a key cause of death among patients with type 2 diabetes [3–5]. Indeed, the entire spectrum of liver disease, abnormal liver enzymes, nonalcoholic fatty liver disease (NAFLD), cirrhosis, hepatocellular carcinoma, and acute liver failure are frequently observed among these patients. Unfortunately, precisely why these associations exist is not well understood.

During the progression of diabetic liver diseases, increasing oxidative stress induced by the hyperglycemia plays several key roles in promoting liver changes or injury [2, 4] and is particularly critical in inducing the cellular dysfunction of liver tissues [6]. Other studies reported that diabetic liver diseases can also be induced by several other factors, for example, activation of the stress signaling pathways, increased cytokine levels, impairment of protective mechanisms, or dysregulation of glucose and lipid metabolism [4, 7, 8]. Curiously, these factors all appear to be driven by hyperglycemia, so the possibility that they may contribute individually or collectively to induce liver injuries exists. To verify if such a possibility exists, pinpointing gene expression changes within the liver may be critical in fully characterizing the underlying mechanisms of diabetes-induced liver diseases. To date, several studies have investigated these changes at the transcriptional level in streptozotocin (STZ) treated rats, db/db mice and hepatic cell lines using qPCR [4], and microarray analysis in Zucker diabetic fatty (ZDF) rats [9, 10], but these models proved to be poorly suited to characterizing the associations in humans. Ultimately, detecting the initial changes to the liver, and thereby improving the outcomes of early therapeutic interventions and preventing organ failure and reducing overall mortality, requires a more sensitive technique for conducting whole genome transcriptional analysis, and such analyses need to be conducted on species more closely related to human beings, such as the nonhuman primates or their close relatives.

Recent advances in both animal models and genomic research may be able to overcome the shortcomings of early studies and allow researchers to draw meaningful, translational results. In terms of technique, RNA sequencing (RNA-seq) has become a powerful tool for conducting transcriptome characterization and gene expression profiling in a high-throughput manner [11, 12], allowing for an unbiased survey of the entire transcriptome and* de novo* assembly that does not require genomic sequences to produce a genome-scale transcription map [11]. With deep coverage and single nucleotide resolution, RNA-seq provides a platform to determine differential expression of genes or isoforms [13] that can also identify novel genes and alternative splicing events (AS) [14], noncoding RNAs [15], and posttranscriptional modifications [16]. Similarly, the increasingly popular use of tree shrews has been shown to overcome some of the inherent limitations in more popular animal models such as mice or rodents. Being a close relative to the primates [17, 18], the tree shrew (*Tupaia belangeri chinensis*) has been successfully used as an animal model of several human diseases, including cancer [19–21], hepatitis B virus (HBV) [22], and metabolism syndromes [23].

To date, the number of RNA-Seq analyses on diabetes is scarce, and studies of diabetes in the tree shrew model are nearly nonexistent. The existing RNA-Seq analyses have primarily focused on the pancreatic islet from both type 1 diabetes and type 2 diabetes subjects [24, 25] and identified putative candidate genes under the proinflammatory cytokines [25] and the saturated fatty acid palmitate [24] and also identified novel mechanisms of *β*-cell dysfunction and death. However, none of these studies has been able to fully explicate the gene expression changes in the liver cells on the early stage of diabetes. The characterization of the transcriptome landscape of the liver may be partially helpful in solving this problem, but without an effective animal model from which results can be accurately extrapolated, such findings would be limited at best. One of our previous studies suggested that streptozotocin (STZ), a glucosamine derivative of nitrosourea and preferentially toxic to pancreatic beta cells, can be used to induce tree shrew diabetes [26]. We found that diabetic tree shrews exhibited higher concentrations of blood glucose, triglyceride (TAG), total cholesterol (CHOL), and impaired glucose intolerance, similar to the symptoms found in humans [26]. Accordingly, applying modern RNA-Seq techniques to a tree shrew type 1 diabetes model may bring promise to better understand the pathogenesis of diabetes-induced liver diseases.

In this study, we first used STZ to induce tree shrews diabetes akin to type 1 diabetes and then took RNA-seq techniques to determine the transcriptome characterization and gene expression profiling of the diabetic and wild type tree shrews. The results of these analyses identified 14,060 and 14,335 genes, respectively, in healthy tree shrews and those with diabetes, with 70 genes differentially expressed between the two groups. Further GO and KEGG annotation revealed that several of the main biological processes of these genes were related to translational processes, steroid metabolic processes, oxidative stress, inflammation, and hypertension, all of which are highly associated with diabetes and its complications.

## 2. Material and Methods

### 2.1. Induction of Diabetes by STZ and Samples Collection

Sixteen male tree shrews captured from the wild near the Animal Centre of Kunming Institute of Zoology and housed in captivity for more than 1 year were collected and then separated into two groups, a control group and a group treated with streptozotocin (STZ) used to induce diabetes which was akin to type 1 diabetes. After overnight fasting (14 hr), body weight and fasting blood glucose were measured prior to injection. The animals in the STZ-induced diabetic group received two intraperitoneal injections of a freshly prepared solution of STZ (Sigma-Aldrich) in 10 mM sodium citrate buffer (PH = 4.2–4.5) at 80 mg/kg of body weight given one week apart while the animals in control group were intraperitoneally injected 10 mM sodium citrate buffer (PH = 4.2–4.5) in comparable amounts. Following injection of either the sodium citrate buffer or STZ, body weight and the fasting blood glucose concentration were measured weekly. All animal care and experimental protocols were approved by the Animal Ethics Committee of Kunming Institute of Zoology, the Chinese Academy of Sciences (Approval number: SYBW20110101-1).

At 4 and 8 weeks after injection, 0.5 mL of femoral vein blood was collected from animals in both groups. The blood samples were treated with heparin lithium salt to anticoagulation, centrifuged at 3000 g for 3 min. The plasma triglycerides (TG), total cholesterol (TC), low-density lipoproteins (LDL), high-density lipoproteins (HDL), and glycated hemoglobin A1c (HbA1c) were assayed by an automatic blood biochemistry analyzer (Ci16200, Abbott, USA) at the First People's Hospital of Yunnan Province, China. The diabetic animals were verified by the concentration of fasting blood glucose and HbA1c. Finally, 5 of the original 8 animals in the STZ-induced diabetic group were regarded as the diabetic animals. At 8 weeks, a total of 6 trees shrews in the control group and 5 diabetic tree shrews were anesthetized, and tissue samples were harvested for further analyses. Each tissue sample was divided into several pieces and snap frozen using liquid nitrogen.

### 2.2. Oral Glucose Tolerance Test (OGTT)

OGTT was followed as previously described [23]. Briefly, tree shrews were fasted overnight (14 hr) prior to the OGTT. Approximately 1 mL of 50% glucose (g/v) base on 3.5 mg/kg (glucose/body weight) was orally administered for the OGTT in all tree shrews. Blood samples were collected from the tail vein and plasma glucose levels were immediately measured at 0, 20, 40, 60, 120, and 180 min after glucose administration using automatic blood glucose test meter (Accu-Chek Performa Blood Glucose Meter, Roche).

### 2.3. RNA Isolation, Library Preparation, and Sequencing

Total RNA was extracted and purified from the frozen liver tissues of each animal using TRIzol (TRansgene) according to the manufacture's protocols. Total RNA was then quantified using the nucleic acid-protein quantitative instrument (Bio-RAD), before the samples from each group were mixed into one sample at the equivalent concentrations. Afterward, the mixed total RNA samples from both the control and diabetic group were packed in dry ice and sent on to Macrogen Millennium Genomics for further library preparation and sequencing. Sequencing was performed using Illumina HiSeq2000 instrument.

### 2.4. Transcriptomic Construction

All reads were mapped to the tree shrew genome (NCBI Ref. database: GCA_000334495.1) [27] using Tophat v2.0.9 [28, 29] with the newest tree shrew annotation file. Reads quality control and statistics were confirmed with FASTQC (http://www.bioinformatics.babraham.ac.uk/projects/fastqc/). Transcripts were assembled and quantified by Cufflinks 2.0.2 [13]. Isoforms assembled by Cufflinks from the two samples were then sent to the Cuffcompare utility, along with the gene annotation file, to generate an integrated combined gtf annotation file. To minimize annotation artifacts, all single exon transcripts mapped to known genes with transcripts lengths shorter than 200 bp were excluded. Transcripts labeled with class code “j” by Cuffcompare software were considered as new isoforms of known genes and added to the original tree shrew annotation file, and the resulting new annotation file was used as the reference file and sent to Cuffcompare utility to generate following events: “annotated exons,” “unknown, generic overlap with reference,” “potentially novel isoforms of genes,” “intergenic transcripts,” “intron retention events,” and “exonic overlap with reference on the opposite strand.” Cuffdiff was used to calculate the FPKM (fragments per kilobase of exon per million fragments mapped) values of each gene in both samples using the new reference file.

### 2.5. Orthology Detection and Gene Functional Enrichment

The Coding Potential Calculator (CPC) [30] was used to determine the coding potential of the transcripts which came from the new loci. Transcript sequences were extracted by gffread, a utility within the Cufflinks package [13]. All probable coding transcript sequences were blasted against the UniProt database using BLASTP with the following parameters and criteria: *E*-value hit filter 1.00*E* − 5, at least 80% sequence identity, and at least 70% query sequence coverage. Database for Annotation, Visualization, and Integrated Discovery (DAVID; http://david.abcc.ncifcrf.gov/) [31] was used to perform gene function enrichment analysis based on GO and KEGG annotation for the significantly expressed genes.

### 2.6. Validation of Differentially Expressed Genes by Quantitative Real Time PCR

Quantitative RT-PCR analysis of selected genes was performed using SYBR green method (TransStart Top Green qPCR SuperMix, TransGen Biotech, Beijing, China) on an ABI PRISM 7900HT (Applied Biosystems, Inc.). The isolated RNA of 3 individuals either from the control group or from the diabetic animals was reverse-transcribed into cDNA using the RT reagent Kit with gDNA Eraser (Takara, DRR047A) in a total volume of 20 *μ*L containing 2 *μ*g of total RNA, following the manufacturer protocol. The cDNA was diluted 20-fold and 2 *μ*L was used as template in subsequent qRT-PCR reactions. Each sample was analyzed in triplicate with the following reaction conditions: 30 seconds at 95°C, followed by 40 cycles of 5 seconds at 95°C, 30 seconds at 60°C, and 20 seconds at 72°C. A dissociation curve was drawn for each primer pair. Relative expression levels of interested genes were determined using 2^−ΔΔCt^ method, and the gene expression levels were normalized to *β-actin* measured in parallel.

### 2.7. Data Availability

The raw dataset have been submitted to NCBI Sequence Read Archive (SRA) under Accession SRX1009946 and SRX1017387, Bioproject: PRJNA282350.

## 3. Results

### 3.1. Diabetic Symptoms of STZ-Induced Tree Shrew

**Figure 1:**
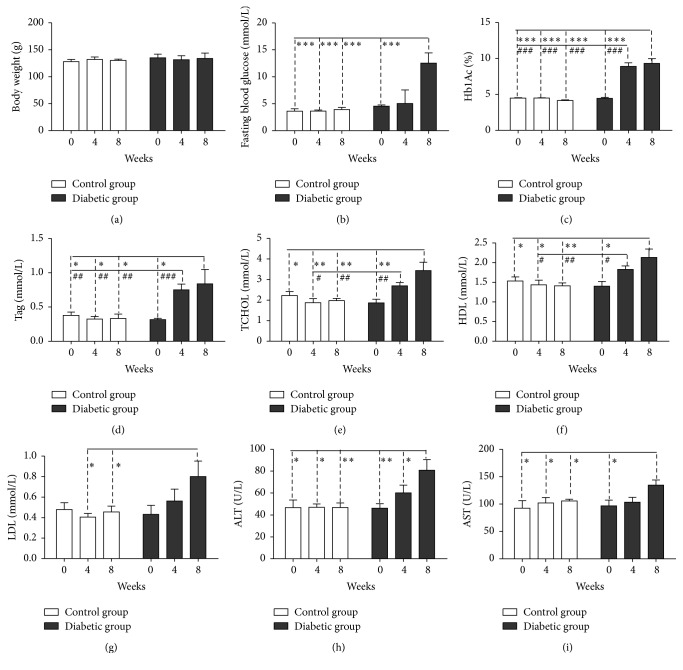
Biochemical parameters in healthy control tree shrews and STZ-induced diabetic tree shrews over 8 weeks. (a) Body weight; (b) plasma concentration of fasting blood glucose; (c) serum concentration of HbA1c; (d) serum concentration of triglycerides (TAG); (e) serum concentration of cholesterol (CHOL); (f) serum concentration of high-density lipoprotein cholesterol (HDL); (g) serum concentration of low-density lipoprotein cholesterol (LDL); (h) serum concentration of alanine aminotransferase (ALT); (i) serum concentration of aspartate aminotransferase (AST). *n* = 6 in control group; *n* = 5 in diabetic group. Significance between two groups. Control group versus 8 weeks of diabetic group: ^*∗*^ *P* < 0.05, ^*∗∗*^ *P* < 0.01, and ^*∗∗∗*^ *P* < 0.001; control group versus 4 weeks of diabetic group: ^#^ *P* < 0.05, ^##^ *P* < 0.01, and ^###^ *P* < 0.001.

**Figure 2:**
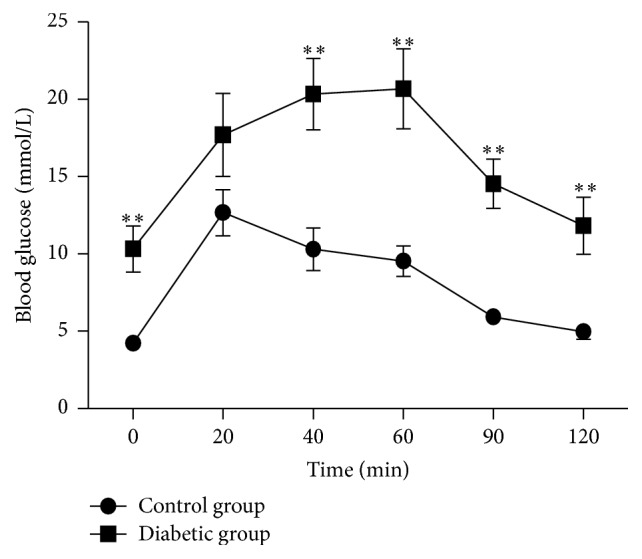
Oral glucose tolerance test (OGTT) in the healthy control group and diabetic group at week 5. *n* = 6 in control group; *n* = 5 in diabetic group. ^*∗∗*^ *P* < 0.01.

The average body weights of tree shrews in the control group and the diabetic group induced by STZ did not change significantly over the 8 wk testing period (Figure 1(a)). Similarly, the fasting blood glucose concentration over the 4 wk period in the diabetic group did not increase significantly (5.03 ± 2.27 mmol/L) as compared with the control group (3.62 ± 0.18 mmol/L) (Figure 1(b)), but the average concentration of the glycosylated hemoglobin (HbA1c) was significantly higher in the diabetic group than that in the control group (*P* < 0.0001) over the 4 wk period (Figure 1(c)). These results indicate a higher postprandial blood glucose concentration in the diabetic group. The oral glucose tolerance test (OGTT) administered at week 5 showed a significant impairment of glucose tolerance in the diabetic group with the significant higher glucose concentration from 20 min to 120 min after glucose administration (Figure 2). At 8 wk, the fasting blood glucose concentration and the value of HbA1c were both significantly higher in the diabetic group (10.42 ± 2.57 mmol/L and 9.31 ± 0.66 mmol/L) than in the control group (3.90 ± 0.38 mmol/L and 4.14 ± 0.09 mmol/L) (Figure 1(b)). These results indicated that STZ-induced tree shrew diabetic model is more likely type 1 diabetes.

The serum total triglycerides (TAG) and total cholesterol (TC) in diabetic animals were both significantly higher than those in the control group animals over the total 8-week period (*P* < 0.05) (Figures 1(d) and 1(e)). Additionally, serum concentrations of the high-density lipoprotein (HDL) and the low-density lipoprotein (LDL) were also highly increased among the diabetic group as compared to the control (Figures 1(f) and 1(g)). To examine the liver injury in the diabetic animals, the serum concentrations of the alanine aminotransferase (ALT) and the aspartate aminotransferase (AST) were detected. The concentrations of ALT and AST increased significantly in the diabetic group at week 8, but not at week 4 (Figures 1(h) and 1(i)).

### 3.2. Mapping and Annotation

**Figure 3:**
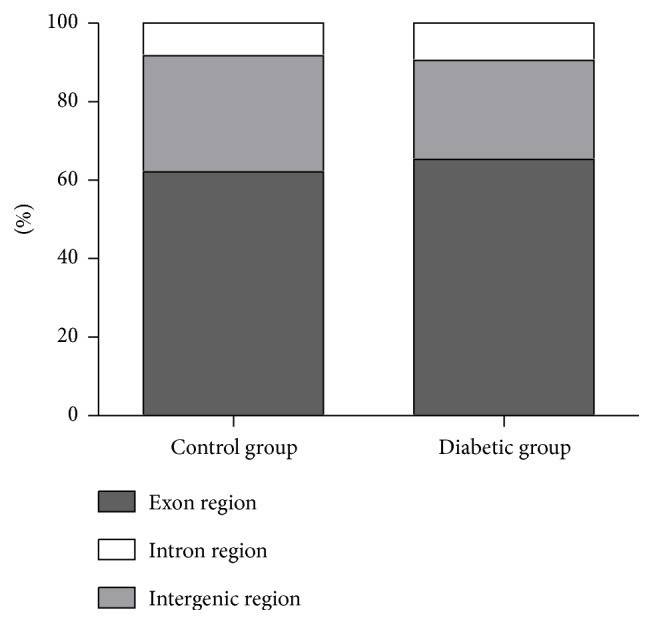
Proportion of reads mapped to the exons, introns, and intergenic regions in liver tissues of the control and diabetic groups.

To obtain comprehensive liver transcripts of the tree shrew and an expression profile in the diabetic tree shrew, total RNAs were isolated from the livers of the control animals and the diabetic animals for RNA-seq using the Illumina instrument. Overall 89,212,358 pair-end 110 bp reads corresponding to more than 18.0 billion base pairs and 73,656,617 pair-end 110 bp reads corresponding to more than 14.8 billion base pairs were obtained in the liver tissues of the control animals and the diabetic animals, respectively. The Tophat software was employed to map the reads against the tree shrew genome [27]. And about 71.5% reads of the control dataset and 75.5% reads of the diabetic dataset, separately, could be mapped to the Chinese tree shrew genome. The mapped reads covered over 90% of the tree shrew genome. Additionally, we calculated the proportion of reads mapped to the exons, introns, and intergenic regions using intersectBed tool from BEDtools package [32]. The highest percentages of reads were mapped to exons (62.1% in control group and 65.3% in diabetic group); and 29.6% of reads in control group and 25.2% of reads in diabetic group were mapped to the intergenic regions; and the lowest percentages of reads (8.3% in control group and 9.5% in diabetic group) fell in the intron regions (Figure 3).

The total numbers of nonredundant assembled transcripts with Cufflinks were 63,748 transcripts from 89,212,358 sequence reads in the control dataset and 55,985 transcripts from 73,656,617 sequence reads in the diabetic dataset. From the Cuffcompare output, these transcripts fall into several categories: annotated exons (35.6% in control group, 31.2% in diabetic group), intron retention events (5.5% in control group, 9.0% in diabetic group), intergenic transcripts (12.5% in control group, 15.5% in diabetic group), potentially novel isoforms of genes (33.6% in control group, 32.0% in diabetic group), pre-mRNA molecules (2.2% in control group, 2.5% in diabetic group), and polymerase run-on fragments (1.7% in control group, 1.7% in diabetic group) (Table 1).

In sum, the total amount of expressed transcripts was 55,976, which generated a total of 24,694 genes in the liver tissues of the control and diabetic groups. A total amount of 7,036 noncoding genes and 2,834 potential coding genes was detected using Coding Potential Calculator (CPC). BLASTP analysis of the 2,834 potential coding genes finally yielded 943 annotated genes.

### 3.3. Gene Expression Analysis

**Figure 4:**
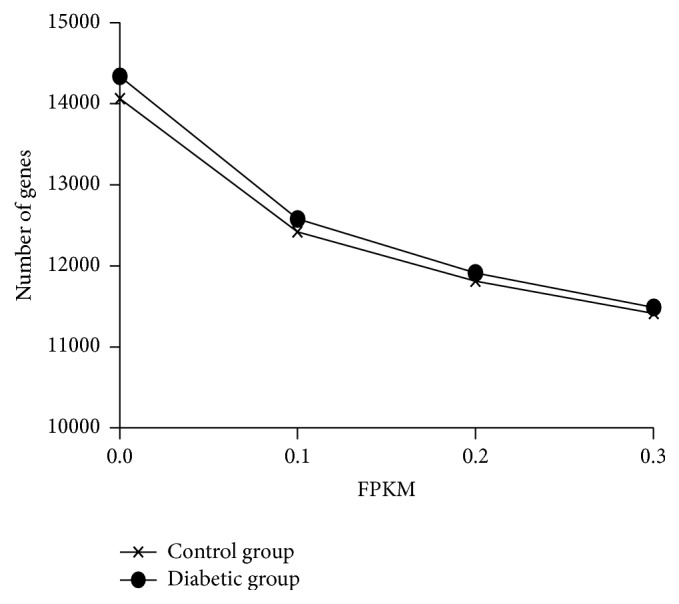
Number of expressed genes with different minimum expression thresholds.

**Figure 5:**
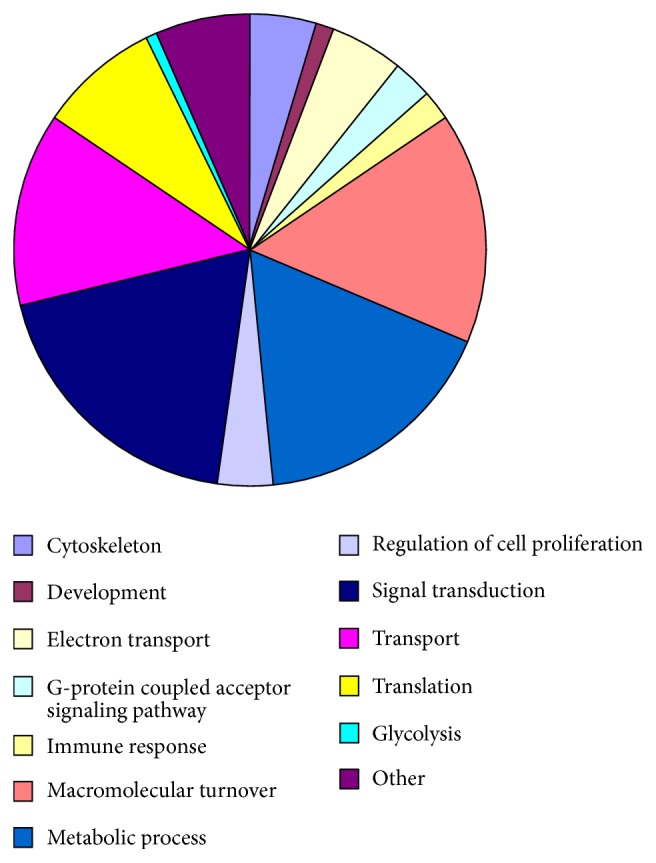
Estimated fractions of cellular transcripts based on gene ontology in the liver tissues of control and diabetic groups. Biological process categories for liver tissues of control tree shrews.

With a total of 14,060 and 14,335 genes, respectively, expressed in the control and diabetic groups, we separately set the threshold value of FPKM to 0.1, 0.2, and 0.3 to balance the numbers of false positives and false negatives and obtained, respectively, 12,418, 11,811, and 11,411 in the control group and 12,578, 11,912, and 11,485 in the diabetic group (Figure 4). Approximately 800 and 600 genes were highly abundant (FPKM > 100) in hepatic tissues of the control and diabetic groups, respectively. To determine the biological functions of genes of the liver cells, we investigated the total cellular mRNA allocating to genes involved in different biological processes in the liver tissues of tree shrew. The categories of biological processes were defined according to [33]. In the liver tissues of tree shrew, three biological processes, including signal transduction, metabolic process, and macromolecular turnover, showed a far higher fraction of transcriptions allocated to genes than other processes (Figure 5), while glycolysis, development, and immune response processes showed a much lower fraction (Figure 5).

### 3.4. Analysis of Differential Gene Expression

**Figure 6:**
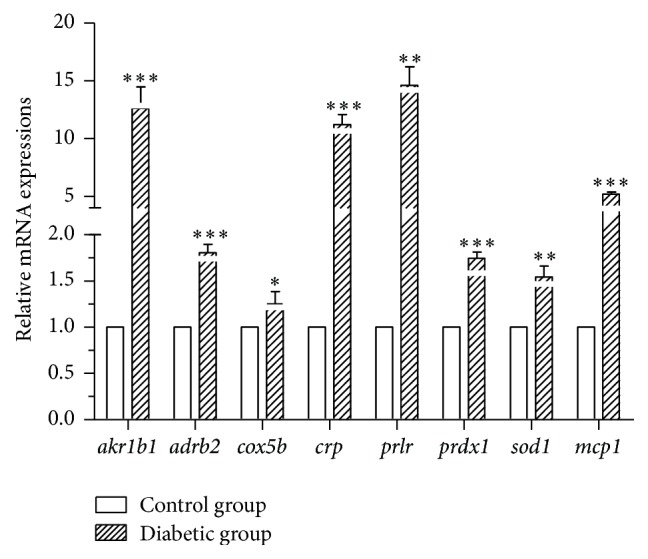
Quantitative-PCR mRNA expression of the randomly selected genes from differentially expressed genes in liver tissues of control and diabetic tree shrews. Significance between two groups; ^*∗*^ *P* < 0.05, ^*∗∗*^ *P* < 0.01, and ^*∗∗∗*^ *P* < 0.001.

Cuffdiff was used to calculate the differentially expressed gene between the control and diabetic groups, yielding 75 differentially expressed genes (Table 2). Among these genes, 23 were upregulated in the diabetic group while the remaining 47 were downregulated (Table 2). To validate the expression level of genes obtained from RNA-Seq, 8 genes (*adrb2*,* akr1b1*,* cox5b*,* crp*,* prlr*,* mcp1*,* prdx1,* and* romo1*) involved in different biological processes were selected for quantitative Real Time PCR (qRT-PCR) (Figure 6). The expression trend of these genes was similar to RNA-Seq platforms.

To explore differences in the biological processes between the healthy and diabetic groups, DAVID [31] was used to perform gene function enrichment analysis based on GO and KEGG annotation for the significantly differentiated genes in both groups (Tables 2 and 3). The main biological functions identified were related to translational process, steroid metabolic process, blood circulation, and so froth, (Table 3). Additionally, several physiological processes and biological processes associated with diabetes, oxidation reduction, electron carrier activity, lipid metabolism, apoptosis regulation, reactive oxygen species response, and inflammatory response were also examined (Table 3).

Our results showed that two genes involved in lipid metabolism were significantly downregulated, potentially due the diabetic status of the tree shrews in the STZ-induced group. The enzymes, acetyl coenzyme A acyltransferase 2 (*acaa2*) (fold difference = −8.01, *P* value = 5.77 × 10^−5^) and the enoyl coenzyme A hydratase, short chain, 1, mitochondrial (*echs1*) (fold difference = −19.90, *P* value = 1.36 × 10^−6^), involved in mitochondrial fatty acid beta-oxidation, showed a markedly lower expression in the diabetic group, implying that the fatty acid breakdown ability was decreased in the diabetic tree shrews. The enzymes* echs1* and* acaa2*, which separately catalyze the second and last steps of the mitochondrial fatty acid beta-oxidation spiral and produce acetyl coenzyme A, were previously reported to candidate genes in type 2 diabetes [34]. Additionally, the downregulation of enzymes* echs1* and* acaa2* inhibits the production of acetyl coenzyme A, leading to the production of ketone bodies and oxidative phosphorylation [34, 35]. We further found that the expression of several genes in the cytochrome P450 family (e.g.,* cyp7a1*,* cyp11a1*,* cyp11b2,* and* cyp17a1*) was upregulated in the diabetic group but not in the control group. These genes appear to be involved in the steroid metabolic process; for example,* cyp7a1* encodes the enzyme cholesterol 7*α*-hydroxylase, which catalyzes the initial step in cholesterol catabolism and bile acid synthesis, while* cyp11a1*,* cyp11b2,* and* cyp17a1* are involved in the process of steroid hormone synthesis. Collectively, this upregulation of genes within the cytochrome P450 family may suggest an increased biosynthesis from cholesterol to the bile acid and steroid hormone.

We also found that several genes associated with the process of oxidative phosphorylation (*cox5a*,* cox5b*,* cox6c*,* mtco1*,* mtco3*,* mtnd4,* and* mtatp6*) were all downregulated in the diabetic group as compared to the control group. Of these genes,* cox5a*,* cox5b,* and* cox6c* are involved in complex IV of the mitochondrial electron transport chain, and downregulation may block the electron transfer through the mitochondrial electron transport chain to generate superoxide. Additionally, within the diabetic group of tree shrews, genes promoting cell apoptosis (*adrb2*,* agtr2*, and* bdkrb2*) were found to be upregulated, while antiapoptotic genes (*npm1*,* dynll1*, and* hspd1*) were downregulated. Likewise, genes associated with the inflammatory process were detected in the RNA-seq data for the diabetic group, including* cxcl13* and* crp.* The* cxcl13* gene, a biomarker of B-cell, displayed a decrease mRNA expression (fold difference = −10.59, *P* value = 4.06 × 10^−5^) in the diabetic group.* crp*, the inflammatory biomarker, showed a significantly higher expression (fold difference = 5.86, *P* value = 1.52 × 10^−4^) in the diabetic group. Finally, we also determined that the genes putatively involved in feeding behavior (*cartpt* and* gal*) were expressed in the diabetic group, but the expression was markedly low among the healthy control group.

## 4. Discussion

### 4.1. Diabetic Dyslipidemia

Among the diabetic tree shrews, we observed significantly higher concentrations of serum TAG, CHOL, HDL, and LDL (Figures 1(d), 1(e), 1(f), and 1(g)), all of which are considered major risk factors for subsequent cardiovascular disease that accompany diabetes mellitus. Earlier reports also found similarly elevated serum TAG, CHOL, and LDL in STZ-induced diabetic rats and mice [36, 37], suggesting that our present tree shrew model induced via STZ replicates the dyslipidemia seen in those test models. In this study, we also observed higher concentration of HDL in the diabetic tree shrews. The increased level of HDL was also commonly observed in type 1 diabetic patients [38, 39]. Some studies reported that the higher HDL cholesterol in type 1 diabetic patients may be related to increased lipoprotein lipase activity and adiponectin [38, 40, 41]. However, STZ-induced rodent diabetes commonly exhibited lower levels of HDL [42, 43]. These results indicate that tree shrew diabetic model might be more appropriate to diabetic research with higher level HDL and to investigate the mechanisms underlying the higher concentration of HDL in type 1 diabetes. Although the precise pathogenesis of diabetic dyslipidemia is not known, a growing number of studies have found that insulin deficiency or insulin resistance and hyperglycemia seem to be key contributors to the disorder [3–7]. Since STZ is preferentially toxic to pancreatic beta cells, the main changes of these upheavals are seen in a reduction of insulin concentration in the blood, suggesting that insulin deficiency and hyperglycemia may also play a critical role during the occurrence of diabetic dyslipidemia within tree shrews, as well as other animal models. More importantly, the serum concentration of AST and ALT increased significantly at week 8 (Figures 1(h) and 1(i)), indicating that diabetic tree shrews appeared to suffer from the same types of liver injury that accompany human diabetes. While this finding may limit some translational aspects of using the tree shrew model, it does provide a novel opportunity to explore the liver changes in diabetic tree shrews that may accompany the early onset of diabetes and help better identify the nature of early changes in the liver tissues that affect diabetic patients with liver diseases.

### 4.2. Transcriptional Profile of Tree Shrew Liver

When applying the threshold value of FPKM to 0.3, our results showed a total of 11,411 and 11,485 genes expressed, respectively, in the control and diabetic groups (Figure 4). On the whole, these numbers are quite similar to those detected in human and mice liver tissues (11,392 and 11,201, resp.) via Ensembl and the proteomic analysis of murine liver cells [33, 44] and in the liver tissues of cows (about 12,500) and pigs (about 10,200) [45, 46]. After examining the categories of biological processes associated with genes expressed in the liver tissues of the tree shrews, we found that three biological processes, signal transduction, metabolic process, and macromolecular turnover, had a far higher fraction of transcriptions allocated to genes than other processes (Figure 5). This transcriptional result is consistent with previous proteomic results, which also found enrichment in the metabolic process and biosynthetic process in the tree shrew liver tissues [44, 47]. Additionally, the fractions of biological processes in the differentiated genes were associated with metabolic processes, translation, electron transport, and inflammatory response. These results are consistent with earlier microarray analyses of liver tissue of ZDF rats, wherein differentially expressed genes were highly associated with metabolic process, signal transduction, inflammatory response [10], and electron transport [9].

### 4.3. Liver Changes during the Early Stage of Diabetes

In the present study we detected a significantly increased expression of the aldose reductase (*akr1b1*), the rate-limiting enzyme of the polyol pathway, which suggests that hyperglycemia may upregulate the expression of aldose reductase (AR) and increase the flux through the polyol pathway in liver tissue of diabetic tree shrews. Accumulation of intracellular sorbitol as a consequence of increased aldose reductase activity was previously implicated in the development of various secondary complications of diabetes. Several studies using experimental animals reported that inhibition of* akr1b1* may be effective in the prevention of some diabetic complications, including kidney, retina, lens, and peripheral neuron tissues [48–50]. However, among animal models with hyperglycemia, increasing of polyol pathway is scarcely observed in the liver tissues. Indeed, a higher expression of* akr1b1* was only observed in liver tissues of STZ-induced type 1 diabetic mice on 4-week period [51], though increased flux of polyol pathway was not detected using microarray analysis in the liver tissues of ZDF rats which are characterized by obesity and type 2 diabetes with a point mutation in the leptin receptor [9, 10]. The reasons for this discrepancy are not entirely clear, but we speculate that the mechanisms used to induce diabetes are different between STZ-induced animal models and the obesity diabetic animal models. Several lines of evidence indicate that the inhibition of AR can prevent oxidative stress induced activation of inflammation that lead to cell death [52], suggesting that oxidative stress may actually be capable of upregulating the expression of AR. Accordingly, the higher expression of AR may be caused by oxidative stress among our diabetic tree shrews.

Impaired mitochondrial oxidative phosphorylation is the primary source of oxidation products (such as reactive oxygen species, ROS), which plays pivotal roles in the genesis of diabetic chronic liver diseases [53, 54]. In this study, three genes involved in the oxidative phosphorylation showed a higher expression differentiation between the control and the diabetic group; within the diabetic group,* cox5a*,* cox5b*, and* cox6c* (complex IV) manifested a significantly lower expression as compared with the control group. This result suggests that the block of electron transfer through the mitochondrial electron transport chain immediately led to the overproduction of superoxide [55]. However, proteomic analysis of liver mitochondria found that most of the genes associated with oxidative phosphorylation were upregulated during the progression of diabetes in GK rats [56]. This discrepancy between the two animal models may be due to either different mechanisms used to induce diabetes or different methods to analyze the data. Likely further replication or comparative studies would be needed to verify this possibility.

Diabetes and hyperglycemia are both known to increase oxidative stress [57, 58]. Convincing experimental and clinical data suggests that the generation of reactive oxygen species increased with diabetes and that the onset of diabetes and its comorbidities and complications are closely associated with oxidative stress [55, 59–63]. High glucose has also been shown to increase oxidative stress, due to combination of increased production of excessive free radicals, ROS along with decreased antioxidant function, which further damaged all components of the cell, including proteins, lipids, and DNA. Here, STZ-induced high glucose levels in the diabetic tree shrews were associated with oxidative stress, consistent with the differential expressed genes of P450 side chain cleavage, P450scc (*cyp11a1*), reactive oxygen species modulator 1 (*romo1*), and peroxiredoxin 1 (*prdx1*). Mitochondrial cytochrome P450 systems have been suggested as a source of mitochondrial ROS production that can play a role in the induction of mitochondrial apoptosis [64]. Moreover, the upregulated expression of* cyp11a1* was purported to potentially indicate an increased ROS in mitochondria of diabetic animals [64]; and the forced expression of* romo1* increased the level of cellular ROS that originate from the mitochondria [65]. Additionally,* prdx1*, ROS scavenging enzymes, was upregulated in diabetic animals, indicating the increasing ability to clean out the free radical, which would alleviate the accumulation of free radical in diabetic liver. Subsequent identification of these genes indicates that oxidative stress in liver tissues occurred during the early stage of diabetes, a finding that may be critical in elucidating the mechanisms that generate the liver changes seen in diabetic patients.

Increased oxidative stress and the simultaneous decline of antioxidant defense mechanisms can lead to inflammation, damage of cellular organelle, and cell death [66–68]. The production of ROS induces inflammatory reactions, and the reverse is also true [66]. The interaction between oxidative stress and inflammatory reactions seems to lead to a vicious circle that eventually induces pathogenesis of diabetic complications. Here, several highly differentiated genes associated with the inflammatory process were detected in the RNA-seq data of the diabetic tree shews, including* cxcl13* and* crp*. The gene* cxcl13*, a biomarker of B-cell, displayed a marked decrease of mRNA expression in the diabetic group, while,* crp*, the inflammatory biomarker, showed significantly higher expression in the same group. Together, these results suggest that inflammatory reactions also occurred in the liver tissues during the early stage of diabetes and that the observed inflammation was mainly induced by ROS. Following this line of evidence, attenuating the oxidative stress and inflammation may prove a useful target for therapeutics aimed at preventing liver injury among diabetes patients.

Mitochondria are thought to be the primary target of oxidative damage, as ROS are generated mainly as by-products of mitochondrial respiration [69]. Several studies also observed mutations in mtDNA and impaired oxidative phosphorylation in diabetic patients [70, 71]. Consistently, the mitochondrial DNA (mtDNA) has been hypothesized to act as a major target for ROS damage [72, 73]. Our analysis of the tree shrew RNA-seq data uncovered several mtDNA genes (*mtco1*,* mtco3*,* mtnd4*, and* mtatp6*) downregulated among the diabetic tree shrews (Table 2). Since these genes are highly associated with the essential components of mitochondria respiration chain [74], the dysfunction of mtDNA genes should impair either the assembly or the function of the respiratory chain. This impairment should in turn trigger further accumulation of ROS, thereby creating a vicious cycle leading to energy depletion in the cell and ultimately cell death [69, 73]. Likewise, mitochondrion dysfunction can result in cell apoptosis [75]. Consistent with this hypothesis, here we observed the upregulation of several genes that promote cell apoptosis (*adrb2*,* agtr2*,* bdkrb2*, and* gdnf*) and a concurrent downregulation of antiapoptotic genes (*npm1*,* dynll1*,* hspd1*, and* prdx1*) among the STZ-induced tree shrews, as compared to the control group. This discrepancy implies that mitochondria may be more vulnerable than other organelles in the hepatic cells during the early stage of diabetes. Furthermore, the dysfunction of mitochondria may further promote inflammation and cell death in the diabetic liver cells. Numerous studies have demonstrated that mitochondrial factors are critical in the development of diabetes [56]. Microarray assay in liver tissues of diabetic ZDF rats and proteomic analysis of liver mitochondria in GK rats previously found impaired oxidative phosphorylation and apoptosis during the development of diabetes [9, 10, 56]. These results collectively indicate that the oxidative phosphorylation of mitochondria likely plays crucial roles in the development of diabetes and in diabetes-induced liver injury.

The liver plays a critical role in regulation of lipid metabolism, accounting for the generation and degradation of fatty acids, steroids, triacylglycerols, cholesterols, bile salts, and so forth, and it is also the central synthetic site of the* de novo* cholesterol biosynthesis in the body. Here, we observed a downregulation of* sc4mol*, a gene involved in the cholesterol biosynthetic process, among diabetic group (Table 2). This result may suggest a decreased synthesis of total cholesterol in the liver. Additionally, we also observed that several genes in cytochrome P450 family (e.g.,* cyp7a1*,* cyp11a1*,* cyp11b2,* and* cyp17a1*) were upregulated in the diabetic group. These genes were previously reported to be involved in the steroid metabolic process, which were in the synthetic processes from cholesterol to the bile acid and steroid hormone. Pullinger et al. [76] demonstrated that the deficiency of* cyp7a1* led to the hypercholesterolemic phenotype among humans, but conversely Pandak et al. [77] found that overexpression of* cyp7a1* in human liver cells might help to lower the concentration of serum cholesterol. By extension, the upregulation of these genes may be helpful in lowering the concentration of serum cholesterol in tree shrew. These results may also hint at a protective mechanism capable of decreasing the concentration of total cholesterol in the liver tissue of the diabetic group, because the high circulation concentration of total cholesterol is the main risk factor for development of cardiovascular diseases. We also observed an increase in the circulation HDL cholesterol particles among the diabetic tree shrews, further hinting at some sort of a protection that prevents the development of atherosclerosis. She et al. [78] previously reported the higher serum HDL in tree shrews after a long period of cholesterol intake, indicating that high serum HDL may play an important role in retarding the development of atherosclerosis, potentially due to the overexcretion of bile acid to degrade large amount of cholesterol in liver of tree shrew. Unfortunately, compared with the hepatic transcriptome profiles of ZDF rats and GK rats, we did not observe the gene changes associated with sucrose/glucose metabolism and tricarboxylic acid cycle, ketone body metabolism, phospholipid/glucolipid metabolism, insulin signaling pathway, ppar signaling pathway, Jak-STAT signaling pathway, and so froth [9, 10, 56]. These discrepancies might be due to the different diabetic phenotypes and different pathognomies. It has reported that STZ-induced type 1 diabetes contributed greatly to the liver dysfunction and cellular damage in rats [79]. Their results are similar to our results in STZ-induced diabetic tree shrews. However, ZDF rat is an obese type 2 diabetes model with a point mutation in the leptin receptor, which makes it an ideal model for studying insulin resistance related to obesity. Additionally, adult GK rats exhibit spontaneous type 2 diabetes with impaired glucose-induced insulin secretion, decreased *β*-cell mass, and hepatic glucose overproduction in the liver [80]. Therefore, genes involved in sucrose/glucose metabolism and tricarboxylic acid cycle, ketone body metabolism, insulin signaling pathway, and ppar signaling pathway could be determined in these models [9, 56]. However, the difference in terms of hepatic gene profiles between type 1 diabetes and type 2 diabetes is still unclear. Further studies still need to investigate this problem.

## 5. Conclusion

In summary, the present study found that STZ can adequately induce diabetes in the tree shrew model, accompanied by higher serum TAG, CHOL, HDL, and LDL. The number of expressed genes and the dominant categories of biological processes in liver transcriptional profile of tree shrew were quite similar to other mammary animals, including humans, pigs, and cows. The differentially expressed genes between the two tested groups of tree shrews also indicated that liver changes accompanied the early stage of diabetes in tree shrew, and hyperglycemia-induced oxidative stress can further lead to higher expression of AR, inflammation, and even cell death in liver tissues.

## Figures and Tables

**Table 1:** Transcripts assembled with Cufflinks and percentage from both the control and diabetic groups.

Code	Control group		Diabetic group	
	Number	%	Number	%
e	1249	2.2	1611	2.5
=	19938	35.6	19904	31.2
x	556	1	657	1
s	2	0	3	0
j	18793	33.6	20376	32
c	3	0	4	0
u	6997	12.5	9910	15.5
p	941	1.7	1101	1.7
.	3251	5.8	3251	5.1
o	1171	2.1	1212	1.9
i	3084	5.5	5719	9
Total	**55985**	**100**	**63748**	**100**

Class codes described by Cuffcompare: “=”: exactly equal to the reference annotation; “c”: contained in the reference annotation; “e”: possible pre-mRNA molecule; “i”: an exon falling into an intron of the reference; “j”: new isoforms; “o”: unknown, generic overlap with reference; “p”: possible polymerase run-on fragment; “s”: an intron of the transfrag overlaps a reference intron on the opposite strand; “u”: unknown intergenic transcript; “x”: exonic overlap with reference on the opposite strand; “.”: tracking file only, which indicates multiple classifications.

**Table 2:** Genes differentially expressed in liver tissues of tree shrew.

ID	Gene name	Gene symbol	Fold changes^*∗*^	Biological process	KEGG pathway
XLOC_001420	Acetyl coenzyme A acyltransferase 2	*Acaa2*	(−) 8.01	Metabolic process	hsa00062:Fatty acid elongation in mitochondria
TSDBG00010707	Beta-2-adrenergic receptor	*Adrb2*	(+) 4.61	G-protein coupled receptor signaling pathway	
TSDBG00019059	Angiotensin II receptor, type 2	*Agtr2*	(+) 693.72	G-protein coupled receptor signaling pathway	
TSDBG00005129	Aldo-keto reductase family 1, member B1	*Akr1b1*	(+) 6.08	Electron transport	
TSDBG00003625	Beta-2-microglobulin	*B2m*	(−) 4.58	Immune response	
TSDBG00006817	Beta-2-bradykinin receptor	*Bdkrb2*	(+) 8.08	Transport	
TSDBG00021527	Calneuron 1	*Caln1*	(+) 18.60	NA	
TSDBG00019726	Cadherin 3	*Cdh3*	(+) 85.20	Cytoskeleton	
TSDBG00017230	Chromogranin A	*Chga*	(+) 47.78	Regulation of blood pressure	
TSDBG00004640	Chromogranin B	*Chgb*	(+) 84.21	Signal transduction	
TSDBG00013910	Cytochrome c oxidase subunit VA	*Cox5a*	(−) 8.05	Electron transport	
TSDBG00011477	Cytochrome c oxidase subunit VB	*Cox5b*	(−) 11.26	Electron transport	
TSDBG00013437	Cytochrome c oxidase subunit VIc	*Cox6c*	(−) 5.81	Electron transport	
TSDBG00018314	C-reactive protein	*Crp*	(+) 5.86	Inflammatory response	
TSDBG00007703	Chemokine (C-X-C motif) ligand 13	*Cxcl13*	(−) 10.59	Immune response	
TSDBG00022385	Cytochrome P450, family 11, subfamily A, polypeptide 1	*Cyp11a1*	(+) 71.64	Electron response	hsa00140:Steroid hormone biosynthesis
TSDBG00016058	Cytochrome P450, family 11, subfamily B, polypeptide 2	*Cyp11b2*	(+) 76.51	Electron response	hsa00141:Steroid hormone biosynthesis
TSDBG00016058	Cytochrome P450, family 11, subfamily B, polypeptide 2	*Cyp11b2*	(+) 68.25	Electron response	hsa00143:Steroid hormone biosynthesis
TSDBG00016479	Cytochrome P450, family 17, subfamily A, polypeptide 1	*Cyp17a1*	(+) 95.77	Electron response	hsa00144:Steroid hormone biosynthesis
TSDBG00010507	Cytochrome P450, family 7, subfamily A, polypeptide 1	*Cyp7a1*	(+) 5.74	Electron response	hsa00144:Steroid hormone biosynthesis
XLOC_008932	Dynein, light chain, LC8-type 1	*Dynll1*	(−) 14.05	Cytoskeleton	
TSDBG00020294	Enoyl CoA hydratase, short chain, 1,	*Echs1*	(−) 19.90	Metabolic process	hsa00062:Fatty acid elongation in mitochondria
TSDBG00018541	Eukaryotic translation elongation factor 1 alpha 1	*Eef1a1*	(−) 5.15	Macromolecular turnover	
XLOC_007804	Ferritin, heavy chain, polypeptide 1	*Fth1*	(−) 12.76	Metabolic process	
TSDBG00003452	G0/G1 switch 2	*G0s2*	(−) 26.68	Signal transduction	
TSDBG00020849	Glial cell derived neurotrophic factor	*Gdnf*	(+) 273.86	Signal transduction	
TSDBG00001160	Hemoglobin, alpha 1	*Hba1*	(−) 6.70	Transport	
TSDBG00005453	Hemoglobin, beta	*Hbb*	(+) 11.90	Transport	
TSDBG00021208	High mobility group nucleosome binding domain 1	*Hmgn1*	(−) 15.39	Cytoskeleton	
XLOC_002257	Heat shock protein 1	*Hspd1*	(−) 5.54	Other	
TSDBG00002459	Kruppel-like factor 6	*Klf6*	(+) 4.90	Macromolecular turnover	
TSDBG00018913	Leukocyte cell-derived chemotaxin 2	*Lect2*	(−) 19.18	Immune response	
TSDBG00013095	Lysozyme	*Lyz*	(−) 10.04	Metabolic process	
TSDBG00002162	Melanocortin 2 receptor	*Mc2r*	(+) 116.92	G-protein coupled receptor signaling pathway	
TSDBG00024122	Mitochondrially encoded ATP synthase 6	*MT-Atp6*	(−) 187.11	Metabolic process	
TSDBG00024207	Mitochondrially encoded cytochrome c oxidase I	*Mt-co1*	(−)	Metabolic process	
TSDBG00024123	Mitochondrially encoded cytochrome c oxidase III	*Mt-co3*	(−)	Metabolic process	
TSDBG00024119	Mitochondrially encoded NADH dehydrogenase 4	*Mt-nd4*	(−)	Metabolic process	
TSDBG00013867	Myomesin 1	*Myom1*	(−) 9.47	Muscle contraction	
TSDBG00014996	Nucleolin	*Ncl*	(−) 22.79	NA	
XLOC_009534	Nucleolar protein interacting with the FHA domain of MKI67	*Nifk*	(−) 20.77	Macromolecular turnover	
XLOC_009785	Nucleophosmin 1	*Npm1*	(−) 23.49	Signal transduction	
TSDBG00009363	Nuclear receptor subfamily 5, group A, member 1	*Nr5a1*	(+) 31.23	Translation	
XLOC_007808	Poly(rC) binding protein 2	*Pcbp2*	(−) 5.67	Immune response	
XLOC_008508	Peroxiredoxin 1	*Prdx1*	(−) 4.40	Response to ROS	
TSDBG00020861	Prolactin receptor	*Prlr*	(+) 8.54	Metabolic process	
TSDBG00023696	Prostate stem cell antigen	*Psca*	(−) 6.50	NA	
TSDBG00017201	Proteasome (prosome, macropain) subunit, alpha type, 6	*Psma6*	(−) 17.58	Other	
XLOC_008889	18S ribosomal RNA	*Rn18s*	(−) 8.17	NA	
XLOC_008890	RNA, 45S pre-ribosomal 5	*Rna45s5*	(−) 6.86	NA	
TSDBG00008648	Reactive oxygen species modulator 1	*Romo1*	(−) 3.84	Response to ROS	
TSDBG00000016	Ribosomal protein L21	*Rpl21*	(−) 16.72	Translation	hsa03013:Ribosome
TSDBG00015118	Ribosomal protein L21	*Rpl21*	(−) 13.55	Translation	hsa03013:Ribosome
TSDBG00021103	Ribosomal protein L21	*Rpl21*	(−) 14.86	Translation	hsa03013:Ribosome
TSDBG00010811	Ribosomal protein L21	*Rpl21*	(−) 78.98	Translation	hsa03013:Ribosome
TSDBG00013239	Ribosomal protein L21	*Rpl21*	(−) 8.21	Translation	hsa03013:Ribosome
TSDBG00008910	Ribosomal protein L22	*Rpl22*	(−) 5.38	Translation	hsa03013:Ribosome
TSDBG00013442	Ribosomal protein L30	*Rpl30*	(−) 5.32	Translation	hsa03013:Ribosome
XLOC_009108	Ribosomal protein L31	*Rpl31*	(−) 11.86	Translation	hsa03013:Ribosome
TSDBG00008508	Ribosomal protein L34	*Rpl34*	(−) 100.22	Translation	hsa03013:Ribosome
XLOC_006890	Ribosomal protein L35a	*Rpl35a*	(−) 16.94	Translation	hsa03013:Ribosome
TSDBG00016157	Ribosomal protein L4	*Rpl4*	(−) 8.55	Translation	hsa03013:Ribosome
TSDBG00001838	Ribosomal protein S24	*Rps24*	(−) 4.50	Translation	hsa03013:Ribosome
TSDBG00006238	Ribosomal protein S24	*Rps24*	(−) 14.75	Translation	hsa03013:Ribosome
TSDBG00013032	Ribosomal protein S26	*Rps26*	(−) 5.76	Translation	hsa03013:Ribosome
XLOC_003904	Methylsterol monooxygenase 1	*Sc4mol*	(−) 4.43	Metabolic process	
TSDBG00006015	Secretogranin II	*Scg2*	(+) 156.25	Signal transduction	
TSDBG00002295	Secretoglobin, family 2A, member 2	*Scgb2a2*	(−) 138.70	NA	
XLOC_008362	Small ubiquitin-like modifier 1	*Sumo1*	(−) 16.29	Regulation of cell proliferation	
TSDBG00017904	Trefoil factor 3	*Tff3*	(−) 15.12	Immune response	
XLOC_010510	Translocase of inner mitochondrial membrane 13	*Timm13*	(−) 5.71	Transport	
TSDBG00009988	Transmembrane protein 252	*Tmem252*	(+) 5.85	NA	
XLOC_004969	Ubiquitin-conjugating enzyme E2D 2A	*Ube2d2a*	(−) 6.67	Other	
TSDBG00019547	Vestigial-like family member 3	*Vgll3*	(+) 17.36	Macromolecular turnover	
XLOC_003709	Vimentin	*Vim*	(−) 13.65	Regulation of cell proliferation	
TSDBG00009142	WAP four-disulfide core domain 2	*Wfdc2*	(+) 8.18	Proteolysis	

^*∗*^Diabetic group/control group; (+): upregulated; (−): downregulated.

**Table 3:** Biological processes and genes likely affected among tree shrews with STZ-induced diabetes.

Function	Genes	*P* value
Translational process	*RPL35A*,* RPS26*,* EEF1A1*,* RPL30*,* RPL31*,* RPL22*,* RPL34*,* RPL21*,* RPL4*,* RPS24*	9.76*E* − 06
Regulation of blood pressure	*ADRB2*,* AGTR2*,* CHGA*,* CYP11B2*,* BDKRB2*,* HBB*	8.53*E* − 04
Steroid metabolic process	*ACAA2*,* CYP17A1*,* PRLR*,* CYP11A1*,* CYP7A1*,* CYP11B2*,* SC4MOL*	1.46*E* − 04
Mitochondrion	*ACAA2*,* CYP11A1*,* CYP11B2*,* ECHS1*,* ROMO1*,* TIMM13*,* COX5A*,* COX5B*,* PRDX1*,* COX6C*,* CYP17A1*,* PSMA6*,* HSPD1*,* MTCO1*,* MTCO3*,* MTND4*,* MTATP6*	0.0019
Electron carrier activity	*CYP7A1*, *CYP17A1*,* CYP11A1*,* CYP11B2*,* AKR1B1*,* COX5A*	0.0019
Oxidation reduction	*CYP7A1*,* CYP17A1*,* CYP11A1*,* CYP11B2*,* AKR1B1*,* COX5A*,* PRDX1*,* FTH1*,* SC4MOL*,* AGTR2*,* GDNF*	0.0036
Regulation of apoptosis	*ADRB2*,* AGTR2*,* PRLR*,* DYNLL1*,* NPM1*,* BDKRB2*,* HSPD1*,* GDNF*,* PRDX1*,* SCG2*	0.0042
Cytochrome c oxidase activity	*COX5A*,* COX5B*,* COX6C*	0.0056
Lipid biosynthetic process	*ACAA2*,* CYP17A1*,* PRLR*,* CYP11A1*,* CYP11B2*,* SC4MOL*	0.0091
Steroid binding	*CYP11A1*,* CYP11B2*,* SCGB2A2*	0.027
Response to reactive oxygen species	*CYP11A1*,* ROMO1*,* PRDX1*	0.036
Inflammatory response	CXCL13, CRP, LYZ, BDKRB2, SCG2	0.040
Fatty acid elongation in mitochondria	*ACAA2*,* ECHS1*	0.046
